# Mir22hg facilitates ferritinophagy-mediated ferroptosis in sepsis by recruiting the m6A reader YTHDC1 and enhancing Angptl4 mRNA stability

**DOI:** 10.1007/s10863-024-10022-1

**Published:** 2024-06-06

**Authors:** Wenlong Deng, Liang Zhong, Shupei Ye, Jiajing Luo, Guobin Ren, Junhao Huang, Xiaolei Zhuang

**Affiliations:** Emergency of Department, SSL Central Hospital of Dongguan City, No.1 Xianglong Road, Shilong Town, Dongguan, 523326 China

**Keywords:** Sepsis, Ferritinophagy, Mir22hg, m6A reader; Angptl4

## Abstract

**Background:**

Ferritinophagy-mediated ferroptosis plays a crucial role in fighting pathogen aggression. The long non-coding RNA Mir22hg is involved in the regulation of ferroptosis and aberrantly overexpression in lipopolysaccharide (LPS)-induced sepsis mice, but whether it regulates sepsis through ferritinophagy-mediated ferroptosis is unclear.

**Methods:**

Mir22hg was screened by bioinformatics analysis. Ferroptosis was assessed by assaying malondialdehyde (MDA), reactive oxygen species (ROS), and Fe^2+^ levels, glutathione (GSH) activity, as well as ferroptosis-related proteins GPX4 and SLC3A2 by using matched kits and performing western blot. Ferritinophagy was assessed by Lyso tracker staining and FerroOrange staining, immunofluorescence analysis of Ferritin and LC-3, and western blot analysis of LC-3II/I, p62, FTH1, and NCOA4. The bind of YTH domain containing 1 (YTHDC1) to Mir22hg or angiopoietin-like-4 (Angptl4) was verified by RNA pull-down and/or immunoprecipitation (RIP) assays.

**Results:**

Mir22hg silencing lightened ferroptosis and ferritinophagy in LPS-induced MLE-12 cells and sepsis mouse models, as presented by the downregulated MDA, ROS, Fe^2+^, NCOA4, and SLC3A2 levels, upregulated GPX4, GSH, and FTH1 levels, along with a decrease in autophagy. Mir22hg could bind to the m6A reader YTHDC1 without affecting its expression. Mechanistically, Mir22hg enhanced Angptl4 mRNA stability through recruiting the m6A reader YTHDC1. Furthermore, Angptl4 overexpression partly overturned Mir22hg inhibition-mediated effects on ferroptosis and ferritinophagy in LPS-induced MLE-12 cells.

**Conclusion:**

Mir22hg contributed to in ferritinophagy-mediated ferroptosis in sepsis via recruiting the m6A reader YTHDC1 and strengthening Angptl4 mRNA stability, highlighting that Mir22hg may be a potential target for sepsis treatment based on ferroptosis.

**Supplementary Information:**

The online version contains supplementary material available at 10.1007/s10863-024-10022-1.

## Introduction

The latest international consensus definition of sepsis is life-threatening organ dysfunction caused by dysregulation of the body’s response to infection (Srzić et al. 2022). Sepsis is the main cause of death due to infection, with the mortality rate of sepsis as high as 20–45% in ICU (Liu et al. 2022a, b; Strandberg et al. 2020). Biomarkers for early recognition of sepsis and targeted therapies for sepsis are lacking (Huang et al. 2019). Presently, there are no effective treatment strategies for sepsis, and supporting therapy in the form of antibiotics and fluid resuscitation remains the mainstay of sepsis treatment (Gavelli et al. 2021). Thus, an aggressive exploration of the pathogenesis of sepsis is important.

An emerging regulatory form of programmed cell death, ferroptosis occurs as a result of the accumulation of intracellular iron-dependent lipid peroxides [2]. Comparing with other cell death pathways such as apoptosis, necrosis and cellular pyroptosis, ferroptosis does not have the morphological characteristics of apoptosis, manifested by iron-induced accumulation of reactive oxygen species (ROS) in lipids and intracellular oxidative stress, resulting in cell death. More recently, ferroptosis has been thought to be closely associated with the severity of sepsis. For instance, the promotion of ferroptosis by IRF7 exacerbated sepsis-induced lung injury through transcriptional activation of SRG3 (Ling et al. 2023). Voltage-dependent anion channel 2 malonylation participated in sepsis-forced myocardial dysfunction via mitochondria-associated ferroptosis (She et al. 2023). Presently, the precise molecular mechanisms of ferroptosis-mediated sepsis have not been elucidated, so further clarification of the role of ferroptosis in sepsis and the associated mechanisms may provide new ideas for the management of sepsis.

Iron ions are metallic elements essential for cells to undergo lipid peroxidation and ferroptosis, so dysfunction or anomalous expression of proteins related to Fe transport and metabolism can regulate ferroptosis. Ferritin autophagy is mediated by nuclear receptor coactivator 4 (NCOA4), in which ferritin is transported to the lysosome or proteasome for degradation, resulting in excessive Fe^2+^ release. Over-production of Fe^2+^ in the cell can directly catalyze lipid ROS generation through the Fenton reaction, resulting in a large accumulation of intracellular lipid ROS, thus triggering ferroptosis. Wang et al. uncovered that ferritinophagy-mediated ferroptosis is one of the key mechanisms of sepsis-induced cardiac injury (Li et al. 2020). Zhang et al. reported that inhibition of ferritinophagy-mediated ferroptosis by YAP1 lessened sepsis-induced acute lung injury (Zhang et al. 2022a, b, c). Although there have been reports to expose that ferritinophagy-mediated ferroptosis exacerbates the severity of sepsis, the molecular mechanisms involved in them are not clear.

During analysis of recurrently dysregulated differentially expressed genes (DEGs) in sepsis, we found that Mir22hg, a long non-coding RNA implicated in ferroptosis, was significantly up-regulated in mouse sepsis models, but whether mir22hg can participate in the progression of sepsis by mediating ferritinophagy-mediated ferroptosis is unclear. This study utilized lipopolysaccharide (LPS) to produce in vivo and in vitro models of sepsis to elucidate whether Mir22hg participates in the severity of sepsis by mediating ferritinophagy-mediated ferroptosis.

## Materials and methods

### Screening of genes related to ferritinophagy-mediated ferroptosis in sepsis

The *GSE165226, GSE179554-6 h* and *GSE179554-12 h* data sets related to mouse sepsis model were selected, and the common deg intersecting genes in these three data sets were screened according to log2FC > 1.4, and the genes involved in sepsis ferritinophagy-mediated ferroptosis were identified.

### Cell culture

Mouse lung epithelial cells MLE-12 (CRL-2110™, ATCC, Manassas, VA, USA) were grown in a specialized medium (CM-0680, Procell, Wuhan, China) containing various components required for its growth. Incubation of the culture was set at 37 °C in a suitable incubator with 5% CO_2_ in air atmosphere. To build sepsis models in vitro, LPS (1 mg/mL, Sigma, St. Louis, Missouri, USA) was used to stimulate MLE-12 cells for 24 h.

### Small interference RNAs (siRNAs), plasmid construction, and transfection

For knocking down Mir22hg in vitro, Mir22hg siRNA (si-Mir22hg) (RiboBio, Guangzhou, China) was transfected into cells with Lipo3000 (Invitrogen, California, USA). Non-targeting siRNA (siNC) (RiboBio) was used as a negative control. YTHDC1 and Angptl4 full-length sequences were PCR-amplified with the primers. The PCR products were cloned into the pcDNA3.1(+) (Invitrogen) to generate YTHDC1 and Angptl4 overexpression plasmids (YTHDC1-OE and Angptl4-OE). For cell transfection, the Lipofectamine 3000 (Invitrogen, Carlsbad, CA, USA) was applied following the instructions for usage provided by the manufacturer.

### Determination of lipid reactive oxygen species (ROS)

BODIPY 581/591 C11 reagent (Invitrogen, California, USA) was used to measure the lipid ROS level. Briefly, after the indicated treatment, cells were dyed with a 5-µM reagent and incubated for 30 min (Zhang et al. 2022a, b, c). Intracellular fluorescence intensity was measured using a flow cytometer (BD Accuri C6 Plus, BD Biosciences, USA).

### Cell viability analysis

Cultivation of MLE-12 cells was carried out in 96-well plates for 48 h at 5 × 10^3^ cells per well. After that, 10 µl of MTT (5 mg/mL) was added to each well and the mixture was incubated for 2 h. Afterwards, DMSO (100 µl per well) was used to replace the MTT reagent for dissolving the crystals. The mixture was shaken for 10 min at room temperature, followed by measurement of absorbance at 490 nm using a microplate reader (Bio-Tek, Vermont, USA).

### Detection of malondialdehyde (MDA), glutathione (GSH), and Fe^2+^ levels

Determination of Fe^2+^ levels in mouse lung tissues and MLE-12 cells was done as per the instructions of the iron assay kit (Sigma). Lipid peroxidation levels and GSH levels were assayed according to the instructions of the MDA Assay Kit (Beyotime) and the GSH Assay Kit (Beyotime).

### Ferroptosis was observed by transmission electron microscopy

The morphology of the cells was directly observed by transmission electron microscopy. The mitochondria became smaller and the mitochondrial membrane density was larger when ferroptosis occurred.

### Cell count and protein concentration detection in BALF

To prepare BALF, the lungs were irrigated with sterile saline 3 times through endotracheal intubation. After centrifugation, the supernatant was stored at -20℃. The precipitated cells were resuspended in saline and the total cell count was measured using a hemocytometer. The percentage of neutrophils was measured by Wright-Giemsa staining. The protein concentration of BALF was determined with BCA protein assay kit (Biyuntian, Jiangsu).

### Western blot

RIPA buffer containing protease inhibitors was used to extract total proteins from homogenized mouse lung tissues and MLE-12. Forty to eighty micrograms of proteins were separated on 10% SDS-PAGE gels and then transferred to polyvinylidene difluoride (PVDF) membranes (Millipore, Burlington, USA). Following blocking, PVDF membranes were incubated for 12 h at 4 °C with the indicated primary antibodies (Abcam, Cambridge, UK): GPX4 (1:1000, #ab125066), SLC3A2 (1:1000, # ab303510), LC-3II/I (0.5 µg/mL, #ab62721), p62 (1:1000, # ab109012), FTH1 (1:1000, #ab183781), NCOA4 (1:1000, #ab314553), YTHDC1 (1:1000, # ab259990), Angptl4 (1:1000, #ab196746) and GAPDH (1:10000, #ab181602). An incubation of the PVDF membrane with goat anti-rabbit secondary antibody (1:2000, #ab6721) was then performed for 50 min. Density of protein bands was quantified using the Alphalmager™ 2000 Imaging System (Alpha Innotech, San Leandro, USA).

### Distribution of ferrous iron in lysosomes

After incubation of MLE-12 cells in 96-well plates, the medium was removed, followed by the addition of 50 nM LysoTracker Green (Beyotime) and 1 mM FerroOrange (Dojindo, Japan). Co-staining was performed for 30 min, followed by replacing the previous solution with fresh PBS (Beyotime). After rinsing with PBS, samples were incubated with secondary antibodies rabbit anti-mouse IgG (Invitrogen) or goat anti-rabbit IgG (Invitrogen). Cell nuclei were stained with DAPI (Beyotime). Cell photographs were taken with an inverted microscope (Olympus, Tokyo, Japan). Quantification was performed by the software Image-Pro Plus 6.0.

### Colocalization of LC3 and ferritin

Transfection of MLE-12 cells with LC3-GFP (Beyotime) for 24 h, followed by stimulation of cells with LPS for 24 h. Cells were permeabilized with 0.03% Triton X-100 (Beyotime) for 60 min, then fixed and blocked with 0.1% BSA (Beyotime) for 1 h. The primary anti-ferritin (Abcam, 1:100, ab75973) and secondary antibodies were applied in turn. MLE-12 cells were evaluated by fluorescence microscopy (Nikon TE-2000, Tokyo, Japan).

For immunofluorescence (IF) staining in lung tissues, the samples were fixed in 4% paraformaldehyde (Beyotime) for 1 h and permeabilized with 0.1% Triton X-100 (Beyotime) for 1 h. After incubation with primary antibodies including anti-LC3 (Affinity Biosciences, 1:100, AF5402) and anti-ferritin (Abcam, 1:100, ab75973), the samples were incubated with secondary antibodies. The samples were imaged with an inverted microscope (Olympus).

### Animal models and grouping

Adenoviruses harboring sh-Mir22hg (Ad-sh-Mir22hg) was obtained from FitGene Biotechnology Co. (Guangzhou, China). Ad containing NC was utilized as the negative control (Ad-NC). All animal experiments were approved by the Institutional Animal Care and Use Committee of SSL Central Hospital of Dongguan City and were performed strictly according to national guidelines. Sepsis mouse models were established as previously described (Xu et al. 2020). After one week of acclimatization feeding, male C57BL/6J mice (6–8 weeks old and 18–21 g weight) were randomly divided into 3 groups, including ctrl + ad-NC, LPS + ad-NC and LPS + ad-sh-Mir22hg. Briefly, mice were subjected to sodium pentobarbital (50 mg/Kg body weight) anesthesia, followed by intraperitoneal injection of 15 mg/Kg LPS (Sigma). An equal amount of PBS was injected intraperitoneally into control mice. Other mice were injected with ad-sh-Mir22hg or ad-NC by the tail vein. All mice were sacrificed and lung tissues were isolated for subsequent analysis.

### Hematoxylin-eosin (HE) staining

Isolated lung samples were fixed with 4% paraformaldehyde for 48 h, followed by dehydration with different concentrations (70-100%) of ethanol solution for 40 min. After paraffin embedding, the samples were cut into 4-mm slices, which were then deparaffinized in xylene, anhydrous ethanol and alcohol. Slides were stained with hematoxylin for 5 min, followed by eosin staining for 1–3 min.

### Quantitative real-time PCR (qPCR)

RNA samples from cells were extracted with TRIzol® reagent (Life technologies, Carlsbad, CA, USA) according to the method provided by the manufacturer. First-strand cDNA was synthesized using Hiscript III reverse transcriptase (Vazyme, Nanjing, China). Relative RNA levels were determined by qPCR on a 7900 Real-Time PCR (Applied Biosystems, USA) system along with ChamQ SYBR qPCR Master Mix (Vazyme). GAPDH was used as an internal control to quantify mRNA levels of genes. Relative levels of mRNA were calculated using the comparative CT (2^−ΔΔCT^) method.

### Subcellular fractionation

Cytoplasmic and nuclear RNA fractions from MLE-12 cells were prepared and collected according to the instructions of the PARIS™ RNA Nuclear/Cytoplasmic Isolation Kit (Life technologies). The levels of Mir22hg were determined by qPCR. U6 was used as a nuclear endogenous control. GAPDH was used as a cytoplasmic control.

### RNA-FISH assays

The cells were fixed with 4% paraformaldehyde and then permeabilized with 0.1% Triton X-100. After washing, the cells were incubated with the probe overnight at 37◦C. DAPI was used for nuclear staining. The FISH probe was prelabeled at the 5 -end with Alexa Fluor 546 NHS ester. Fluorescence was detected using a DMI8 microscope (Leica).

### In vitro pull-down assay with biotinylated RNA

The m6A sites in Angptl4 was mutated (mut). Full-length Mir22hg (F1), truncated Mir22hg (F2-F9), full-length Angptl4 wild type (WT) and its m6A mut sequence were biotinylated in vitro using the AmpliScribe T7-flash Biotin-RNA Transcription Kit according to the manufacturer’s instructions. Protein samples of MLE-12 cells were incubated with streptomycin beads (Invitrogen) preincubated with a biotinylated probe for 1 h. The complexes were precipitated, washed four times, and analyzed by western blot.

### RNA-binding protein immunoprecipitation (RIP)

Using the Magna RIP Kit (Millipore), we performed RIP assays by following the manufacture’s instructions. Cells lysed with RIP lysis buffer, and the extracts were mixed with magnetic beads and incubated with primary antibodies against IgG (ab172730, 1:100, Abcam) or YTHDC1 (ab259990, 1:50, Abcam) for 2 h at 4 °C. RNA was then purified with TRIzol (Life technologies) and quantified by qPCR analysis.

### Methylated RNA immunoprecipitation (MeRIP)

After 48 h transfection, total RNA (300ug/time) was extracted and MeRIP experiment was performed using the Magna MeRIP Kit (Millipore, Burlington, MA, USA) according to the manufacture’s instructions. In brief, the cells were lysed in a MeRIP lysis buffer, and magnetic beads were conjugated with a mouse antibody against m6A (ab208577, 1:50, Abcam) or IgG (ab172730, 1:100, Abcam) for 2 h at 4 °C. Subsequently, the obtained RNA was assessed by qPCR.

### RNA stability assay

Following overnight incubation of cells in 6-well plates, 5 µg/mL of actinomycin D (Sigma) was added for inhibiting gene transcription. After that, RNA was then extracted and subjected to qPCR analysis.

### Statistical analysis

Results are reported as mean ± standard deviation (SD). All experiments were repeated at least three times, each time with three technical replicates. Significant differences between two groups that conformed to normally distributed data were tested using Student’s t-test. For comparisons of more than two groups, ANOVA with Tukey’s post hoc test was used to calculate p-values. *P* < 0.05 was considered statistically significant, and *p* < 0.01 and *p* < 0.001 were considered statistically highly significant.

## Results

### Mir22hg knockdown curtailed LPS-induced ferroptosis

**Fig. 1:**
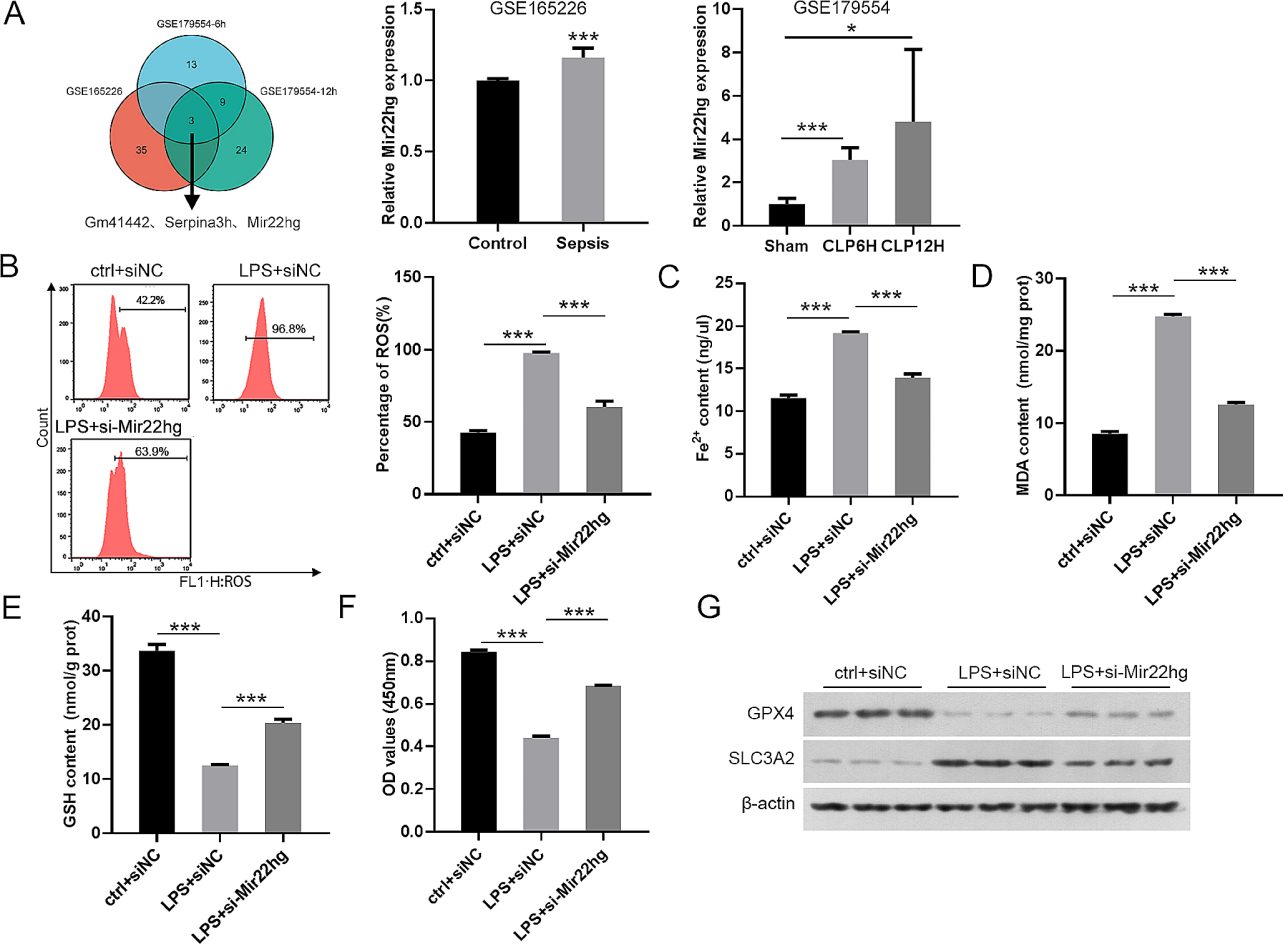
Inhibition of Mir22hg impaired LPS-induced ferroptosis. (**A**) Schematic representation of DEGs intersected in three datasets GSE165226, GSE179554-6 h, and GSE179554-12 h (log_2_FC > 1.4). (**B**) Representative images and quantitative results of the fluorescent probe (DCFH-DA) for intracellular lipid ROS generation in different groups (ctrl + siNC, LPS + siNC, and LPS + si-Mir22hg). (**C**-**F**) The contents of Fe^2+^, MDA, and GSH were determined using the indicated commercial kits. (**F**) Cell viability was determined by MTT assays. (**G**) Western blot was used for analysis of GPX4 and SLC3A2 protein levels. ^*^p < 0.05, ^***^p < 0.001

To identify DEGs in sepsis, we analyzed the GSE165226, GSE179554-6 h, and GSE179554-12 h datasets associated with mouse sepsis models. A total of 3 DEGs were intersected in these three datasets (log_2_FC>1.4), including Gm41442, Serpina3h, and Mir22hg (Fig. 1A), only Mir22hg has been reported to be associated with ferroptosis and autophagy (Wen et al. 2020). So, we explored whether Mir22hg is involved in sepsis by regulating ferritinophagy-mediated ferroptosis.

To verify the role of Mir22hg, we silenced Mir22hg in the MLE-12 cells using si-Mir22hg (Supplementary Fig. 1A). Mir22hg silencing attenuated LPS-induced the levels of lipid ROS, Fe^2+^ and MDA, (Fig. 1B-D). Moreover, Mir22hg knockdown increased cell activity and GSH levels decreased by LPS treatment (Fig. 1E, F). LPS-stimulated MLE-12 cells appeared to have a marked decrease in GPX4 protein levels and a distinct increase in SLC3A2 protein levels, but these alterations were reversed upon Mir22hg silencing (Fig. 1G). There was no difference in GPX4 and SLC3A2 mRNA expression among all groups (Supplementary Fig. S1B, C). Collectively, LPS-induced MLE-12 cell ferroptosis could be partially neutralized by knocking down Mir22hg.

### Mir22hg insufficiency abrogated LPS-induced ferritinophagy

**Fig. 2:**
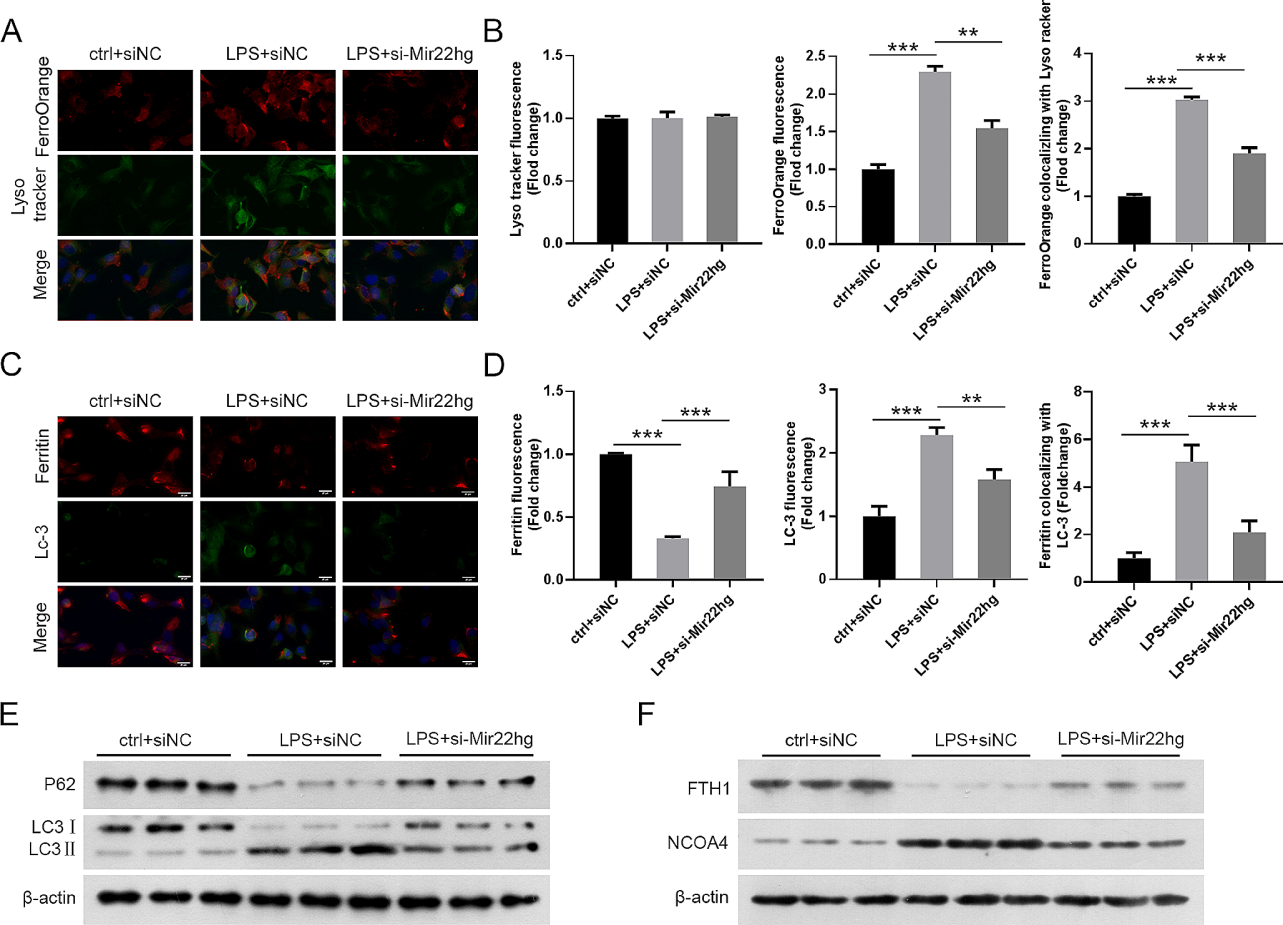
Silencing of Mir22hg repressed LPS-induced ferritinophagy. (**A**, **B**) LysoTracker (green) and FerroOrange (red) were shown by immunofluorescence imaging in MLE-12 cells at different groups (ctrl + siNC, LPS siNC, and LPS + si-Mir22hg). (**C**, **D**) Representative immunofluorescence images of ferritin (red) with LC3 (green). (**E**, **F**) Protein levels of LC-3II/I, p62, FTH1, and NCOA4 were shown by western blot. ^**^p < 0.01, ^***^p < 0.001

To investigate whether knockdown of Mir22hg is associated with ferritinophagy in LPS-induced MLE-12 cells, we assayed lysosomal activity. Co-localization of LysoTracker (green) and FerroOrange (red) was enhanced after LPS stimulation, indicating the presence of large amounts of Fe^2+^ in lysosomes. However, the levels of free Fe^2+^ in lysosomes were attenuated in Mir22hg-deficient cells in response to LPS stimulation (Fig. 2A, B). Stimulation with LPS decreased ferritin levels and enhanced LC3 levels in MLE-12 cells. The colocalized fluorescence of Ferritin with LC3 was intensified on exposure to LPS, while Mir22hg silencing attenuated the intensity of LC3 and Ferritin colocalization (yellow dots) in LPS-stimulated cells (Fig. 2C, D). We found that LC3II /I and NCOA4 protein levels were elevated in LPS-exposed MLE-12 cells, while FTH1 and p62 levels were decreased. However, silencing of Mir22hg partially reversed the LPS-induced alterations in the above-mentioned proteins (Fig. 2E, F). Taken together, Mir22hg silencing decreased LPS-induced ferritinophagy in MLE-12 cells.

### Mir22hg silencing weakened LPS-induced sepsis by inhibiting ferroptosis and ferritinophagy in mouse models

**Fig. 3:**
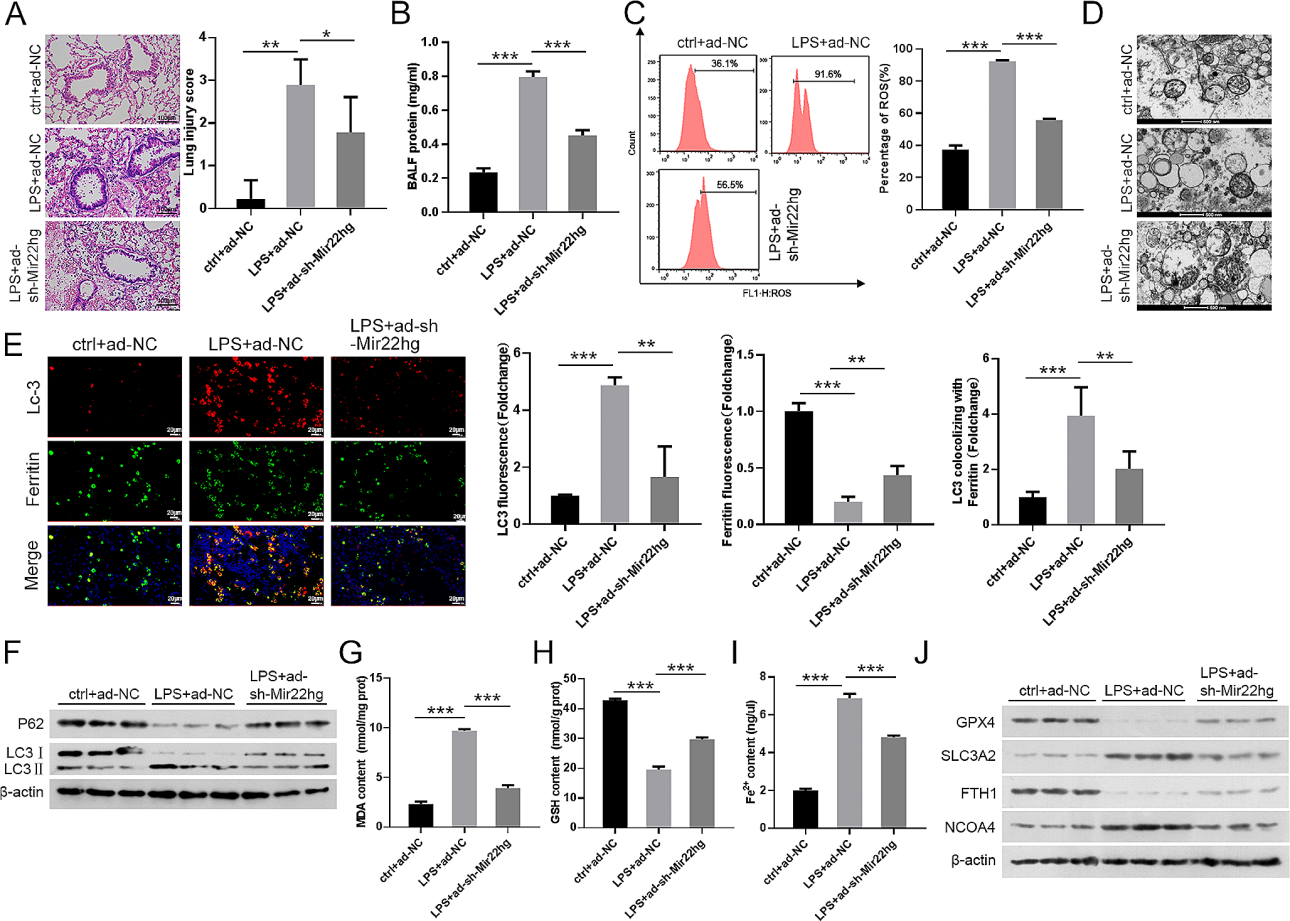
Suppression of Mir22hg attenuated LPS-induced sepsis by repressing ferroptosis and ferritinophagy. (**A**) Representative images of HE staining of lung samples from mice treated with ctrl + ad-NC, LPS + ad-NC, or LPS + ad-sh-Mir22hg. (**B**) BALF protein levels in lung tissues. (**C**) The levels of lipid ROS were analyzed by flow cytometry. (**D**) Transmission electron microscopy for ferroptosis. (**E**) Representative images of ferritin (red) and LC3 (green) in lung tissues. (**F**) Western blot analysis of LC-3II/I and p62 protein levels in lung tissues. (**G**-**I**) The levels of MDA (**G**), GSH (**H**), and Fe^2+^ (**I**) in lung tissues were assessed using the indicated commercial kits. (**J**) Relative protein levels of GPX4, SLC3A2, FTH1, and NCOA4 in lung tissues were detected by western blot. ^*^p < 0.05, ^**^p < 0.01, ^***^p < 0.001

To further validate the function of Mir22hg in sepsis, we induced sepsis in mice with LPS, followed by injection of ad-NC or ad-sh-Mir22hg via tail vein. As shown in Supplementary Fig. 1D, ad-sh-Mir22hg inhibited LPS-induced the expression of Mir22hg in lung tissues. Furthermore, LPS treatment caused significant pathological changes, including alveolar hemorrhage and massive inflammatory cell infiltration, while the introduction of ad-sh-Mir22hg attenuated the above pathological changes (Fig. 3A). Moreover, LPS treatment caused a significant protein increase in lung BALF, while ad-sh-Mir22hg injection resulted in a decrease in BALF (Fig. 3B). The ferroptosis of the cells was observed by transmission electron microscopy, and the photographs showed that the normal mitochondrial morphology was observed in the control group, and knockdown of Mir22hg inhibited LPS-induced mitochondrial damage (Fig.3D). LPS stimulation decreased ferritin levels and increased LC3 levels in lung tissues, as well as enhanced the colocalized fluorescence of ferritin and LC3, whereas silencing of Mir22hg reversed these changes (Fig. 3E). Meanwhile, we observed elevated LC-3II/I;protein expression and reduced p62 protein levels in lung tissues of LPS-induced mouse origin, whereas inhibition of Mir22hg reversed the changes in LC-3II/I and p62 protein levels (Fig. 3F). In response to LPS stimulation, lipid ROS, MDA and Fe^2+^ levels were elevated and GSH levels were reduced in lung tissues of mice, but Mir22hg knockdown was able to partially override these alterations (Fig. 3C, G-I). Also, LPS stimulation caused up-regulation of SLC3A2 and NCOA4 protein levels as well as down-regulation of GPX4 and FTH1 protein levels in mouse lung tissues, but knockdown of Mir22hg partially overrode these changes (Fig. 3J). To sum up, Mir22hg knockdown attenuated LPS-induced sepsis in mouse models.

### Mir22hg could interact with YTHDC1

**Fig. 4:**
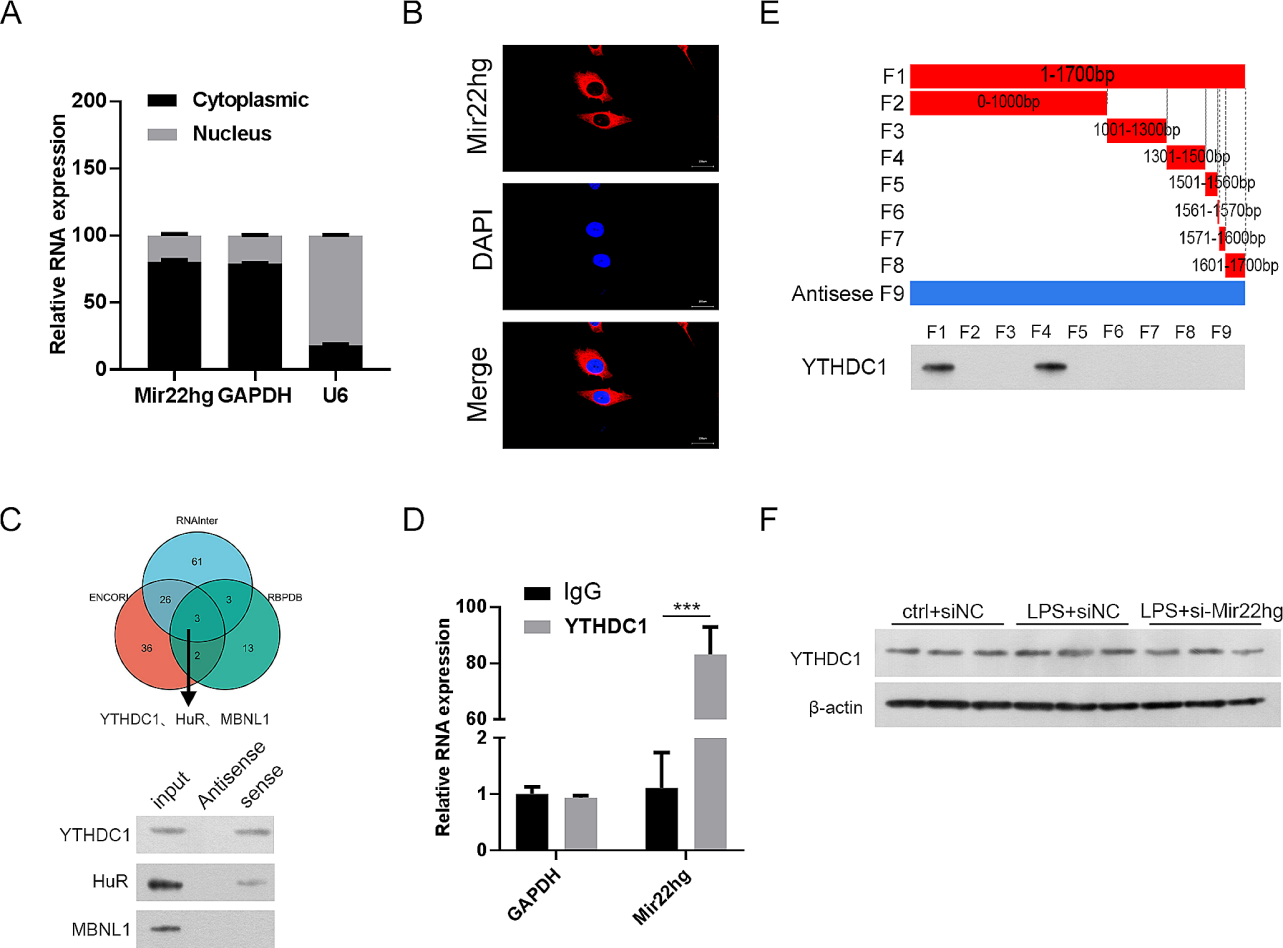
Mir22hg could bind to YTHDC1. (**A**) Nuclear-cytoplasmic fractionation assays determined Mir22hg expression in MLE-12 cells with LPS stimulation, with U6 (nucleus) and GAPDH (cytoplasm) as positive controls. (**B**) RNA-FISH analyzed the subcellular localization of Mir22hg in LPS-stimulated MLE-12 cells. (**C**, **D**) RNA pulldown and RIP assays were carried out to assess the interaction between Mir22hg and YTHDC1. (**E**) A series of truncated sequences of Mir22hg were used in RNA pull down assays to identify the core region of Mir22hg required for interaction with YTHDC1. (F) Effects of Mir22hg on YTHDC1 protein levels were determined by western blot in MLE-12 cells at different groups (ctrl + siNC, LPS + siNC, and LPS + si-Mir22hg). ^***^p < 0.001

Next, the molecular mechanisms involved in Mir22hg were further investigated. Subcellular fractionation and RNA-FISH assays revealed that Mir22hg was mainly distributed in the cytoplasm (Fig. 4A, B, Supplementary Fig. S1E), signifying that Mir22hg may perform its functions through multiple potential mechanisms. lncRNA has been reported to play an important role in sepsis by interacting with RNA binding proteins (RBPs) (Miao and Tu 2023). Using the bioinformatics databases RBPD, RNAInter, and ENCORIB, we found that ELAVL1, MBNL1, and YTHDC1 might bind to Mir22hg. The interaction between YTHDC1 and Mir22hg was assessed by RNA pulldown and RIP assays, and an essential binding between them was discovered (Fig. 4C-D). To further identify the binding region of YTHDC1 on Mir22hg, we prepared a series of Mir22hg gene truncations. RNA pulldown presented that the full length of Mir22hg (F1) and its truncation F4 could bind to YTHDC1, while the other truncations did not show significant binding ability (Fig. 4E), suggesting that the nucleotides at positions 1301–1500 of Mir22hg are the key sites for binding to YTHDC1. Next, we further clarified whether YTHDC1 expression was regulated by Mir22hg. No distinct changes in YTHDC1 expression were observed in MLE-12 cells with and without Mir22hg knockdown in response to LPS stimulation (Fig. 4F), implying that YTHDC1 might be recruited by Mir22hg to modulate downstream targets. Collectively, YTHDC1 might be recruited by Mir22hg in LPS-stimulated MLE-12 cells.

### Mir22hg/YTHDC1 complex enhances Angptl4 mRNA stability via an m6A-dependent manner

**Fig. 5:**
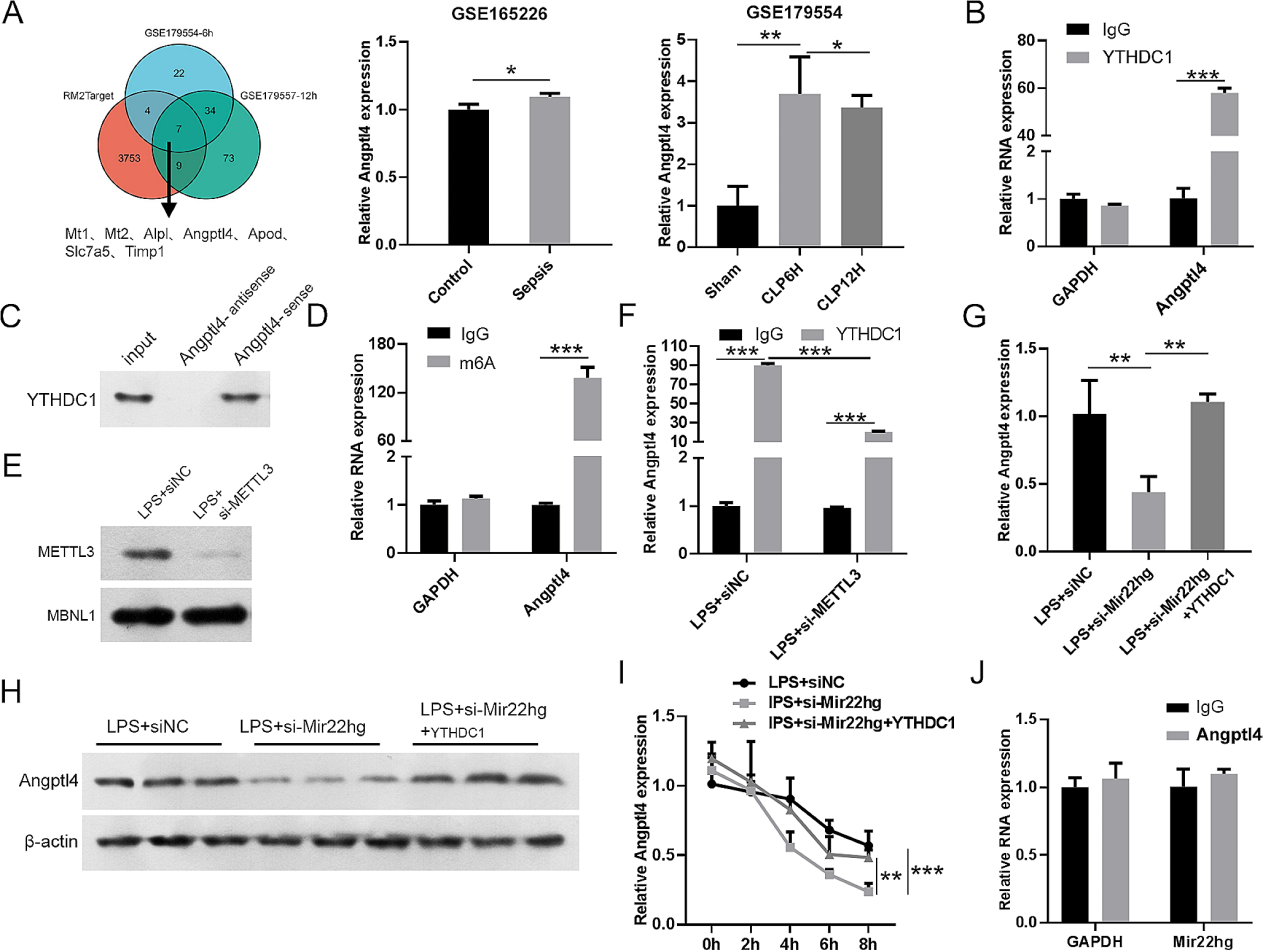
The Mir22hg/YTHDC1 complex stabilized Angptl4 mRNA in an m6a-dependent manner. (**A**) Schematic representation of Angptl4 intersected in three datasets GSE165226, GSE179554-6 h, and GSE179554-12 h. (**B**, **C**) Interaction between Angptl4 and YTHDC1 in MLE-12 cells with LPS stimulation was validated by RNA pulldown and RIP assays. (**D**) MeRIP assays in LPS-stimulated MLE-12 cells confirming that Angptl4 exhibits m6A modification. (**E**) The protein expression of METTL3 in the LPS-stimulated MLE-12 cells transfected with si-NC or si-METTL3. (**F**) Angptl4 enrichment in METTL3-knockdown cells was evaluated by RIP assay (using IGF2BP2-specific antibody). (**G**, **H**) Relative levels of Angptl4 mRNA and protein in MLE-12 cells at different groups (ctrl + siNC, LPS + siNC, and LPS + si-Mir22hg) were assessed by qPCR and western blot. (**I**) Analysis of Mir22hg expression in MLE-12 cells at different groups (ctrl + siNC, LPS + siNC, and LPS + si-Mir22hg) treated with actinomycin D (5 mg/mL) for the indicated periods. (**J**) Estimation of the interaction between Mir22hg and Angptl4 in LPS-stimulated MLE-12 cells was performed by RIP assays. ^*^p < 0.05, ^**^p < 0.01, ^***^p < 0.001

The intersection of databases RM2Target, GSE179554-6 h, and GSE179554-12 h presented 7 genes (Mt1, Mt2, Alpl, Angptl4, Apod, Slc7a5, and Timp1) that may bind towards YTHDC1 in sepsis (Fig. 5A). Among them, Angptl4 has been reported to promote ferroptosis and autophagy (Zhan et al. 2022), and knockdown of Angptl4 can inhibit sepsis-induced acute lung injury (Sun et al. 2023). Given that YTHDC1 is an m6A reader, can maintain the stability and nuclear export of m6A-modified RNAs by binding m6A sites (Wang et al. 2023a, b). Therefore, we investigated whether YTHDC1 regulates Angptl4 by an m6A dependent manner. RIP and RNA pulldown assays verified the interaction between Angptl4 and YTHDC1 in LPS-stimulated MLE-12 cells (Fig. 5B, C). We also observed that Angptl4 mRNA was enriched in the anti-m6A group, as determined by meRIP assay (Fig. 5D). Further, mutating the m6A motif in Angptl4 decreased the binding of YTHDC1, as detected by RNA pulldown assay (Supplementary Fig. S1G). m6A modifications are deposited via the m6A methyltransferase complex (MTC, also known as the m6A writer). METTL3 serves as the central catalytic subunit of the MTC and plays a crucial role in m6A modification (Yang et al. 2022). Thus, METTL3 was used to investigate whether the interaction between YTHDC1 protein and Angptl4 mRNA is dependent on m6A modifications. METTL3 siRNA was used to knockdown METTL3 in LPS-stimulated MLE-12 cells (Fig. 5E). Subsequently, the enrichment of Angptl4 mRNA in the YTHDC1 or m6A immunoprecipitation was abolished upon METTL3 knockdown (Fig. 5F, Supplementary Fig. 1F). To examine whether the mRNA stability of Angptl4 is indeed affected by Mir22hg/YTHDC1 axis, LPS-stimulated MLE-12 cells transfected with siMir22hg or siMir22hg + YTHDC1 were treated with actinomycin D. As shown in Fig. 5G, H,Mir22hg suppression reduced Angptl4 mRNA and protein levels in LPS stimulation cells, but these effects were partly reversed after YTHDC1 overexpression. Moreover, silencing of Mir22hg significantly reduced the stability of Angptl4 mRNA, whereas its stability was relatively enhanced by YTHDC1 overexpression (Fig. 5I). In addition, Mir22hg failed to enrich in the complexes precipitated by the anti-Angptl4 antibody (Fig. 5J), implying that Angptl4 could not bind to Mir22hg directly. Clustering together, these results suggested that Mir22hg recruited YTHDC1 to reinforce the stability of Angptl4 mRNA in a m6A-dependent manner.

### Angptl4 overexpression partially reversed Mir22hg knockdown-mediated effects on LPS-induced ferroptosis

**Fig. 6:**
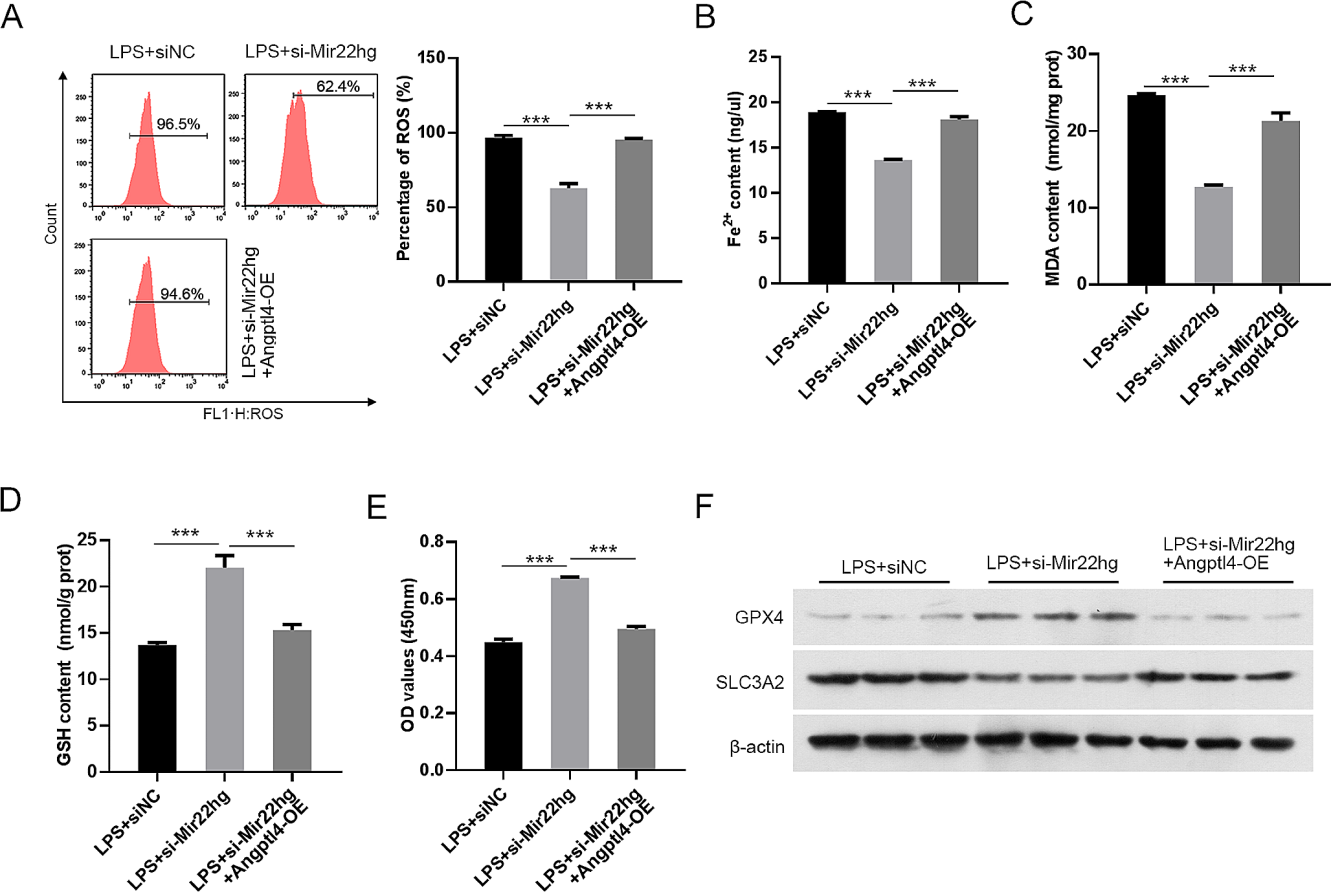
Mir22hg mediated ferroptosis by Angptl4 in LPS-induced MLE-12 **cells.** (**A**) Analysis of lipid ROS levels in MLE-12 cells at different groups (LPS + siNC, LPS + si-Mir22hg, LPS + si-Mir22hg + Angptl4-OE) by flow cytometry. (**B**-**D**) The indicated commercial kits were used for assessment of the contents of Fe^2+^, MDA, and GSH. (**E**) MTT assays were utilized to analyze the viability of MLE-12 cells. (**F**) Western blot was carried out for analysis of GPX4 and SLC3A2 protein levels. ^***^p < 0.001

To further clarify whether Angptl4 is a functional downstream target of Mir22hg, we performed rescue experiments. As shown in Supplementary Fig. S1H, overexpression of Angptl4 increased the expression of Angptl4 in the LPS-induced MLE-12 cells with Mir22hg knockdown. Furthermore, Mir22hg silencing-mediated the inhibitory effect on lipid ROS, Fe^2+^ and MDA levels in LPS-stimulated MLE-12 cells were reversed partly by Angptl4 overexpression (Fig. 6A-C). Moreover, Mir22hg silencing-mediated elevated cellular activity and GSH levels were attenuated by up-regulation of Angptl4 in LPS-stimulated MLE-12 cells (Fig. 6D, E). In addition, the elevated GPX4 protein levels and the reduced SLC3A2 protein levels in LPS-stimulated MLE-12 cells mediated by Mir22hg repression were impaired following Angptl4 overexpression (Fig. 6F). These outcomes manifested that Mir22hg mediated LPS-induced MLE-12 cell ferroptosis by mediating Angptl4 expression.

### Angptl4 up-regulation partially reversed Mir22hg knockdown-mediated effects on LPS-induced ferritinophagy

**Fig. 7:**
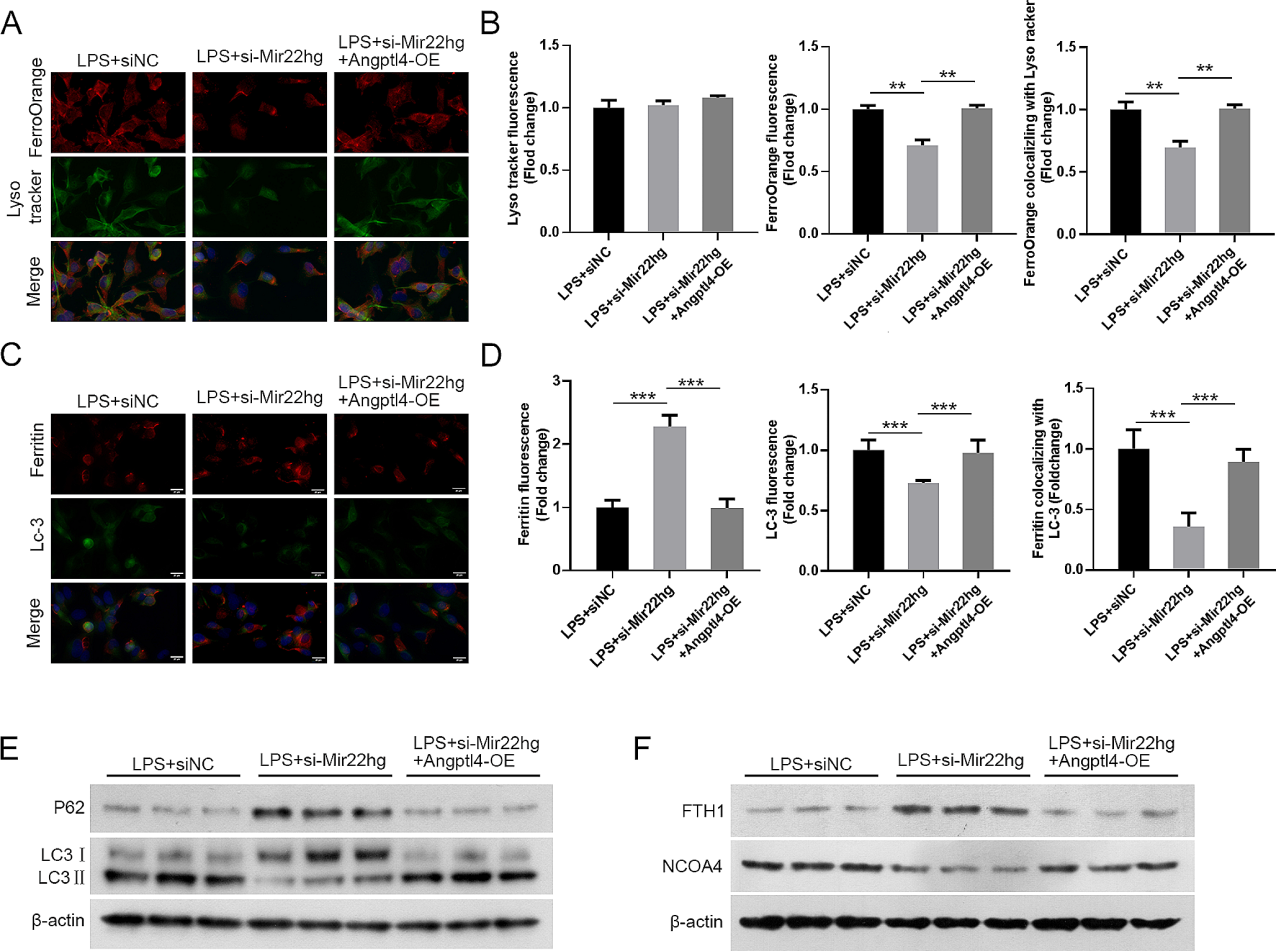
Mir22hg regulated ferritinophagy through Angptl4 in LPS-induced MLE-12 cells. (**A**, **B**) Representative images of LysoTracker (green) and FerroOrange (red) in MLE-12 cells at different groups (LPS + siNC, LPS + si-Mir22hg, LPS + si-Mir22hg + Angptl4-OE). (**C**, **D**) Representative images of ferritin (red) with LC3 (green). (**E**, **F**) Detection of LC-3II/I, p62, FTH1, and NCOA4 protein levels were conducted by western blot. ^**^p < 0.01, ^***^p < 0.001

We further addressed whether Angptl4 is connected to Mir22hg knockdown-mediated effects on LPS-induced ferritinophagy. The results presented that Angptl4 overexpression partly overtured Mir22hg suppression-mediated inhibiting effects on Fe^2+^ levels in MLE-12 with LPS treatment in lysosome (Fig. 7A, B). Mir22hg down-regulation-urged elevation of ferritin levels and reduction of LC3 levels in MLE-12 cells treated with LPS were weakened after Angptl4 overexpression for Ferritin autophagy colocalization (Fig. 7C, D). In addition, elevation of Angptl4 impaired Mir22hg inhibition-mediated effects on LC3II/I, NCOA4, FTH1, and p62 levels in LPS-induced MLE-12, as validate by western blot (Fig. 7E, F). In summary, Mir22hg mediated LPS-induced MLE-12 cell ferritinophagy by Angptl4.

## Discussion

In this study, we investigated the effect of Mir22hg on sepsis progression mediated by ferroptosis through in vivo animal models and in vitro cellular models. Here, we discovered that Mir22hg knockdown attenuated LPS-induced sepsis by decreasing ferritinophagy-mediated ferroptosis in LPS-induced mouse sepsis models as well as in LPS-induced MLE-12 cells. Mechanistically, Mir22hg enhanced Angptl4 mRNA stability by recruiting YTHDC1. This study provided a reference for targeting ferritinophagy toward sepsis treatment.

Emerging findings have suggested that lncRNAs participate in the progression of various diseases by mediating ferroptosis (Lin et al. 2022; Liu et al. 2022a, b; Tang et al. 2022), including sepsis (Wei et al. 2022). Wei et al. revealed that lncRNA NEAT1 augmented sepsis-associated encephalopathy by facilitating ferroptosis by regulating TFRC and GOT1 expression levels (Wei et al. 2022). Mir22hg, located at 17p13.3, is a heterozygous deletion or hypermethylated chromosomal region (Zhang et al. 2018). Available studies have shown that Mir22hg plays a cancer inhibitory role in gastric cancer (Li and Wang 2019), hepatocellular carcinoma (Su et al. 2018), lung cancer (Wang et al. 2023a, b), and colorectal cancer (Xu et al. 2020), whereas it promotes tumor progression in esophageal cancer (Su et al. 2019). Ge et al. exposed that Mir22hg exacerbated cardiomyocyte injury via miR-9-3p/SH2B3 axis in response to oxygen-glucose deprivation and reoxygenation induction (Ge et al. 2022). Also, Mir22hg could activated the AKT signaling via deregulation of PTEN, thereby promoting osteogenic differentiation (Jin et al. 2020). A previous study revealed that Mir22hg is highly expressed in a mouse model of sepsis (Kopczynski et al. 2021), but studies confirmed the action of Mir22hg in sepsis have not been discovered. Here, we used LPS to induce MLE-12 cells and mice for mimicking sepsis-induced lung injury models in vitro and in vivo. The results showed that the effects of LPS-induced massive accumulation of Fe^2+^, marked elevation of lipid ROS and MDA, and obvious reduction of GSH content were attenuated by Mir22hg silencing in LPS-induced cells and animal models, suggesting that Mir22hg exerted a promoting effect on LPS-induced ferroptosis.

When cells undergo autophagy, autophagosomes mainly use NCOA4 as a receptor for autophagy-dependent ferritin degradation (Santana-Codina et al. 2021). By overexpressing NCOA4, they inhibit the expression of FTH1, which in turn promotes the release of iron, enhances the Fenton response, and induces ferroptosis in the cells (Guo et al. 2021). As an upstream regulatory target in the ferroptosis cascade reaction, ferritinophagy can mediate the amount of Fe^2+^ release by affecting the levels of autophagy-related proteins NCOA4 and FTH1, thereby modulating the onset of ferroptosis (Xiu et al. 2022; Fang et al. 2021). In the current study, we observed that the alterations in LPS-induced autophagy enhancement, up-regulation of NCOA4 protein, and down-regulation of FTH1 protein in LPS-induced cells and animal models were attenuated after knockdown of Mir22hg, indicating that Mir22hg might promote ferroptosis by promoting ferritinophagy in sepsis-induced lung injury.

The functions of most lncRNAs are closely related to the RNA-binding proteins (RBPs) that bind to each other (Yao et al. 2022). Our findings revealed that Mir22hg was located in both the nucleus and cytoplasm of MLE-12 cells. Mir22hg was predicted to bind to YTHDC1 using online databases and verified by RNA pulldown and RIP assays. Moreover, Mir22hg could bind to YTHDC1 but did not regulate its expression in LPS-induced MLE-12 cells. YTHDC1 is the only YTH family methyl-binding protein located in the nucleus among m6A modifications (Xiao et al. 2016), and it regulates a variety of metabolic responses by affecting the nuclear location, stability, attenuation, and splicing of RNAs, thereby influencing the development of diseases (Kasowitz et al. 2018; Roundtree et al. 2017; Widagdo et al. 2022). YTHDC1 can interact with lncRNAs to perform different biological functions and regulate the occurrence and development of diseases (Jin and Liu 2023; Wu et al. 2023). Tang et al. found that LINC00857 interacted with YTHDC1 to regulate SLC7A5 and increase the stability of SLC7A5 mRNA, thus promoting the proliferation and migration of colorectal cancer cells (Tang et al. 2021). In addition, YTHDC1 was reported to be a regulator of ferroptosis by modulating the stability of FSP1 mRNA (Yuan et al. 2023). Also, YTHDC1 improved ferroptosis in response to sleep deprivation by activating the SIRT1/NRF2/HO1 pathway (Chen et al. 2023). YTHDC1 targeted SQSTM1 in diabetic skin to regulate autophagy (Liang et al. 2022). Therefore, we hypothesized that Mir22hg might regulate other gene expression by binding to YTHDC1, thereby regulating ferritinophagy.

Angptl4, a member of the angiopoietin-like protein family (Angptl), is widely distributed in the vascular system (Waschki et al. 2016) and visceral adipose tissues (Janssen et al. 2018). Angptl4 is involved in the regulation of tumor formation, vascular permeability, lipid metabolism, energy balance, wound healing, inflammation and oxidoreductive processes (Aryal et al. 2019; Fernández-Hernando and Suárez 2020). Microarray identification revealed that Angptl4 may be a potential target gene for the treatment of burn sepsis (Xu et al. 2015). Angptl4 knockdown attenuated sepsis-induced lung injury through blocking the activation of the NF-κB pathway and hindering macrophage M1 polarization (Sun et al. 2023). Angptl4 mediated radio-resistance of lung cancer by inhibiting ferroptosis (Zhang et al. 2022a, b, c). Angptl4 was revealed to be a directly regulated target gene of TAZ, and it sensitized ferroptosis by activating NOX2 (Yang et al. 2020). Within the current study, we demonstrated that Angptl4 could interact with YTHDC1. Mir22hg/YTHDC1 enhances the mRNA stability of Angptl4 through an m6A-dependent manner. Furthermore, Angptl4 overexpression partially counteracted Mir22hg knockdown-mediated effects on LPS-induced ferroptosis and ferritinophagy.

**Fig. 8:**
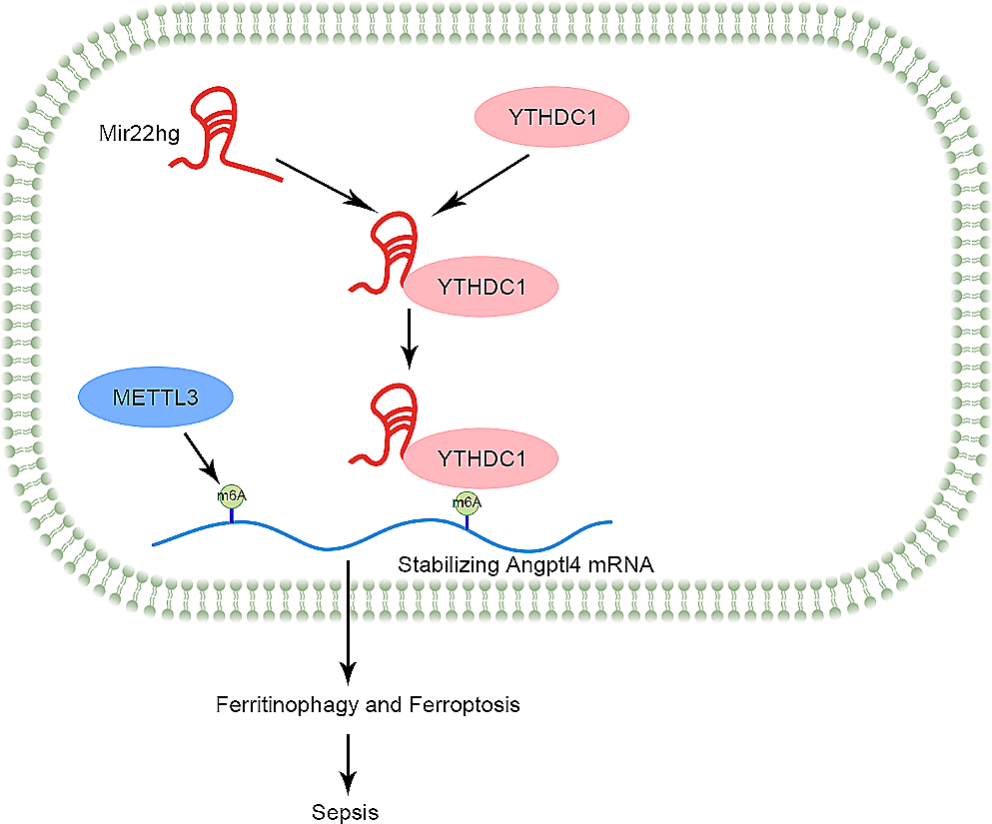
An illustration of the molecular mechanism in sepsis

In conclusion, Mir22hg was able to recruit the m6A reader YTHDC1 to stabilize Angptl4 mRNA expression, thereby promoting ferritinophagy to accelerate sepsis progression (Fig. 8). Unfortunately, the detailed mechanisms associated with the m6A modifications involved in this study were not explored, which will be further explored in the future. This study highlighted that Mir22hg may be a potential target for sepsis treatment by interfering with ferritinophagy.

### Electronic supplementary material

Below is the link to the electronic supplementary material.

Supplementary Material 1

Supplementary Material 2

Supplementary Material 3

## Data Availability

The datasets of this study are available from the corresponding author upon request..

## References

[CR47] Aryal B, Price NL, Suarez Y, Fernández-Hernando C (2019). ANGPTL4 in Metabolic and Cardiovascular Disease. Trends Mol Med.

[CR43] Chen J, Xiao L, Chen Y, Li W, Liu Y, Zhou Y, Tan H (2023). YT521-B homology domain containing 1 ameliorates mitochondrial damage and ferroptosis in sleep deprivation by activating the sirtuin 1/nuclear factor erythroid-derived 2-like 2/heme oxygenase 1 pathway. Brain Res Bull.

[CR33] Fang Y, Chen X, Tan Q, Zhou H, Xu J, Gu Q (2021). Inhibiting ferroptosis through disrupting the NCOA4-FTH1 Interaction: a new mechanism of action. ACS Cent Sci.

[CR48] Fernández-Hernando C, Suárez Y (2020). ANGPTL4: a multifunctional protein involved in metabolism and vascular homeostasis. Curr Opin Hematol.

[CR5] Gavelli F, Castello LM, Avanzi GC (2021). Management of sepsis and septic shock in the emergency department. Intern Emerg Med.

[CR27] Ge Y, Liu L, Luo L, Fang Y, Ni T (2022) MIR22HG Aggravates oxygen-glucose deprivation and reoxygenation-induced cardiomyocyte injury through the miR-9-3p/SH2B3 Axis. *Cardiovascular therapeutics* ; 2022:7332298 10.1155/2022/7332298 PMC9173999 35692373

[CR31] Guo W, Zhao Y, Li H, Lei L (2021). NCOA4-mediated ferritinophagy promoted inflammatory responses in periodontitis. J Periodontal Res.

[CR4] Huang M, Cai S, Su J (2019) The pathogenesis of Sepsis and potential therapeutic targets. Int J Mol Sci ; 20(21) 10.3390/ijms20215376 PMC6862039 31671729

[CR46] Janssen AWF, Katiraei S, Bartosinska B, Eberhard D, van Willems K, Kersten S (2018). Loss of angiopoietin-like 4 (ANGPTL4) in mice with diet-induced obesity uncouples visceral obesity from glucose intolerance partly via the gut microbiota. Diabetologia.

[CR39] Jin Z, Liu Y (2023). The m6A reader YTHDC1-mediated lncRNA CTBP1-AS2 m6A modification accelerates cholangiocarcinoma progression. Heliyon.

[CR28] Jin C, Jia L, Tang Z, Zheng Y (2020). Long non-coding RNA MIR22HG promotes osteogenic differentiation of bone marrow mesenchymal stem cells via PTEN/ AKT pathway. Cell Death Dis.

[CR36] Kasowitz SD, Ma J, Anderson SJ, Leu NA, Xu Y, Gregory BD (2018). Nuclear m6A reader YTHDC1 regulates alternative polyadenylation and splicing during mouse oocyte development. PLoS Genet.

[CR29] Kopczynski M, Rumienczyk I, Kulecka M, Statkiewicz M, Pysniak K, Sandowska-Markiewicz Z et al (2021) selective extracellular signal-regulated kinase 1/2 (ERK1/2) inhibition by the SCH772984 compound attenuates in vitro and in vivo inflammatory responses and prolongs survival in murine sepsis models. 22(19):10204 10.3390/ijms221910204 PMC8508766 34638546

[CR19] Li H, Wang Y (2019). Long noncoding RNA (lncRNA) MIR22HG suppresses gastric cancer progression through attenuating NOTCH2 signaling. Med Sci Monitor: Int Med J Experimental Clin Res.

[CR8] Li N, Wang W, Zhou H, Wu Q, Duan M, Liu C (2020). Ferritinophagy-mediated ferroptosis is involved in sepsis-induced cardiac injury. Free Radic Biol Med.

[CR44] Liang D, Lin WJ, Ren M, Qiu J, Yang C, Wang X (2022). M(6)a reader YTHDC1 modulates autophagy by targeting SQSTM1 in diabetic skin. Autophagy.

[CR14] Lin Z, Song J, Gao Y, Huang S, Dou R, Zhong P (2022). Hypoxia-induced HIF-1α/lncRNA-PMAN inhibits ferroptosis by promoting the cytoplasmic translocation of ELAVL1 in peritoneal dissemination from gastric cancer. Redox Biol.

[CR6] Ling X, Wei S, Ling D, Cao S, Chang R, Wang Q, Yuan Z (2023). Irf7 regulates the expression of Srg3 and ferroptosis axis aggravated sepsis-induced acute lung injury. Cell Mol Biol Lett.

[CR2] Liu YC, Yao Y, Yu MM, Gao YL, Qi AL, Jiang TY (2022). Frequency and mortality of sepsis and septic shock in China: a systematic review and meta-analysis. BMC Infect Dis.

[CR15] Liu Y, Zhang Z, Yang J, Wang J, Wu Y, Zhu R et al (2022b) lncRNA ZFAS1 positively facilitates endothelial ferroptosis via miR-7-5p/ACSL4 axis in diabetic retinopathy. *Oxidative medicine and cellular longevity* ; 2022:9004738 10.1155/2022/9004738 PMC9453005 36092160

[CR23] Miao R, Tu J (2023). LncRNA CDKN2B-AS1 interacts with LIN28B to exacerbate sepsis-induced acute lung injury by inducing HIF-1α/NLRP3-mediated pyroptosis. Kaohsiung J Med Sci.

[CR37] Roundtree IA, Luo GZ, Zhang Z, Wang X, Zhou T, Cui Y et al (2017) YTHDC1 mediates nuclear export of N(6)-methyladenosine methylated mRNAs. eLife ; 6 10.7554/eLife.31311 PMC5648532 28984244

[CR30] Santana-Codina N, Gikandi A, Mancias JD (2021). The role of NCOA4-Mediated Ferritinophagy in Ferroptosis. Adv Exp Med Biol.

[CR7] She H, Tan L, Du Y, Zhou Y, Guo N, Zhang J (2023). VDAC2 malonylation participates in sepsis-induced myocardial dysfunction via mitochondrial-related ferroptosis. Int J Biol Sci.

[CR1] Srzić I, Nesek Adam V, Tunjić Pejak D (2022). SEPSIS DEFINITION: WHAT’S NEW IN THE TREATMENT GUIDELINES. Acta Clin Croatica.

[CR3] Strandberg G, Walther S, Agvald Öhman C, Lipcsey M (2020). Mortality after severe Sepsis and septic shock in Swedish intensive care units 2008-2016-A nationwide observational study. Acta Anaesthesiol Scand.

[CR20] Su W, Feng S, Chen X, Yang X, Mao R, Guo C (2018). Silencing of long noncoding RNA MIR22HG triggers cell Survival/Death signaling via oncogenes YBX1, MET, and p21 in Lung Cancer. Cancer Res.

[CR26] Su W, Guo C, Wang L, Wang Z, Yang X, Niu F (2019). LncRNA MIR22HG abrogation inhibits proliferation and induces apoptosis in esophageal adenocarcinoma cells via activation of the STAT3/c-Myc/FAK signaling. Aging.

[CR13] Sun B, Bai L, Li Q, Sun Y, Li M, Wang J (2023). Knockdown of angiopoietin-like 4 suppresses sepsis-induced acute lung injury by blocking the NF-κB pathway activation and hindering macrophage M1 polarization and pyroptosis. Toxicol vitro: Int J Published Association BIBRA.

[CR41] Tang S, Liu Q, Xu M (2021). LINC00857 promotes cell proliferation and migration in colorectal cancer by interacting with YTHDC1 and stabilizing SLC7A5. Oncol Lett.

[CR16] Tang R, Wu Z, Rong Z, Xu J, Wang W, Zhang B et al (2022) Ferroptosis-related lncRNA pairs to predict the clinical outcome and molecular characteristics of pancreatic ductal adenocarcinoma. Brief Bioinform ; 23(1) 10.1093/bib/bbab388 34553745

[CR21] Wang S, Wang Y, Li S, Nian S, Xu W, Liang F (2023). Long non-coding RNA MIR22HG inhibits the proliferation and migration, and promotes apoptosis by targeting microRNA-9-3p/ SOCS1 axis in small cell lung cancer cells. Mol Biol Rep.

[CR24] Wang X, Chen Q, Bing Z, Zhou S, Xu Z, Hou Y (2023). Low expression of m6A reader YTHDC1 promotes progression of ovarian cancer via PIK3R1/STAT3/GANAB axis. Int J Biol Sci.

[CR45] Waschki B, Kirsten AM, Holz O, Meyer T, Lichtinghagen R, Rabe KF (2016). Angiopoietin-like protein 4 and cardiovascular function in COPD. BMJ Open Respir Res.

[CR17] Wei XB, Jiang WQ, Zeng JH, Huang LQ, Ding HG, Jing YW (2022). Exosome-derived lncRNA NEAT1 exacerbates Sepsis-Associated Encephalopathy by promoting ferroptosis through regulating miR-9-5p/TFRC and GOT1 Axis. Mol Neurobiol.

[CR11] Wen J, Liu J, Jiang H, Wan L, Xin L, Sun Y (2020). lncRNA expression profiles related to apoptosis and autophagy in peripheral blood mononuclear cells of patients with rheumatoid arthritis. FEBS open bio.

[CR38] Widagdo J, Anggono V, Wong JJ (2022). The multifaceted effects of YTHDC1-mediated nuclear m(6)a recognition. Trends Genet.

[CR40] Wu C, Cui J, Huo Y, Shi L, Wang C (2023). Alternative splicing of HOXB-AS3 underlie the promoting effect of nuclear m6A reader YTHDC1 on the self-renewal of leukemic stem cells in acute myeloid leukemia. Int J Biol Macromol.

[CR35] Xiao W, Adhikari S, Dahal U, Chen YS, Hao YJ, Sun BF (2016). Nuclear m(6)a reader YTHDC1 regulates mRNA splicing. Mol Cell.

[CR32] Xiu Z, Zhu Y, Han J, Li Y, Yang X, Yang G (2022). Caryophyllene Oxide induces Ferritinophagy by regulating the NCOA4/FTH1/LC3 pathway in Hepatocellular Carcinoma. Front Pharmacol.

[CR49] Xu X, Shi Z, Hu J, Yuan B, Huang H, Fang H (2015). Identification of differentially expressed genes associated with burn sepsis using microarray. Int J Mol Med.

[CR22] Xu J, Shao T, Song M, Xie Y, Zhou J, Yin J (2020). MIR22HG acts as a tumor suppressor via TGFβ/SMAD signaling and facilitates immunotherapy in colorectal cancer. Mol Cancer.

[CR51] Yang WH, Huang Z, Wu J, Ding CC, Murphy SK, Chi JT (2020). A TAZ-ANGPTL4-NOX2 Axis regulates ferroptotic cell death and Chemoresistance in epithelial ovarian Cancer. Mol cancer Research: MCR.

[CR25] Yang J, Qian X, Qiu Q, Xu L, Pan M, Li J (2022). LCAT1 is an oncogenic LncRNA by stabilizing the IGF2BP2-CDC6 axis. Cell Death Dis.

[CR34] Yao ZT, Yang YM, Sun MM, He Y, Liao L, Chen KS, Li B (2022). New insights into the interplay between long non-coding RNAs and RNA-binding proteins in cancer. Cancer Commun (London England).

[CR42] Yuan S, Xi S, Weng H, Guo MM, Zhang JH, Yu ZP et al (2023) YTHDC1 as a tumor progression suppressor through modulating FSP1-dependent ferroptosis suppression in lung cancer. Cell Death Differ 10.1038/s41418-023-01234-w PMC10733405 37903990

[CR12] Zhan W, Tian W, Zhang W, Tian H, Sun T (2022). ANGPTL4 attenuates palmitic acid-induced endothelial cell injury by increasing autophagy. Cell Signal.

[CR18] Zhang DY, Zou XJ, Cao CH, Zhang T, Lei L, Qi XL (2018). Identification and functional characterization of long non-coding RNA MIR22HG as a tumor suppressor for Hepatocellular Carcinoma. Theranostics.

[CR9] Zhang J, Zheng Y, Wang Y, Wang J, Sang A, Song X, Li X (2022). YAP1 alleviates sepsis-induced acute lung injury via inhibiting ferritinophagy-mediated ferroptosis. Front Immunol.

[CR10] Zhang J, Zheng Y, Wang Y, Wang J, Sang A, Song X (2022). YAP1 alleviates sepsis-induced acute lung injury via inhibiting ferritinophagy-mediated ferroptosis. Front Immunol.

[CR50] Zhang Y, Liu X, Zeng L, Zhao X, Chen Q, Pan Y (2022). Exosomal protein angiopoietin-like 4 mediated radioresistance of lung cancer by inhibiting ferroptosis under hypoxic microenvironment. Br J Cancer.

