# Supplementary figures and images for: Mir22hg facilitates ferritinophagy-mediated ferroptosis in sepsis by recruiting the m6A reader YTHDC1 and enhancing Angptl4 mRNA stability
Source: J Bioenerg Biomembr. 2024 Jun 6;56(4):405–18. doi: 10.1007/s10863-024-10022-1 (PMC11217081; doi:10.1007/s10863-024-10022-1)

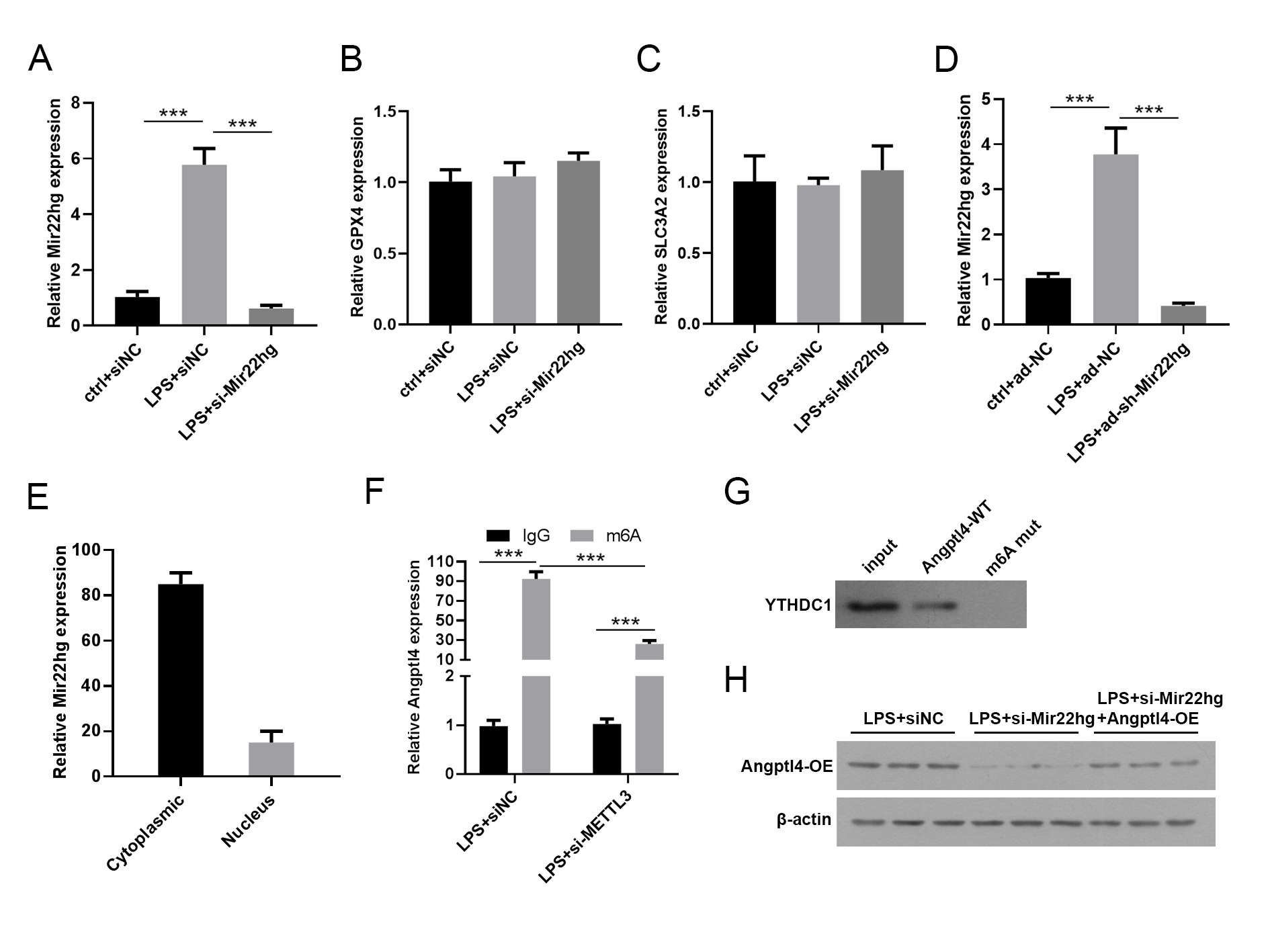

Supplement: Supplementary file 1 — Supplementary Material 1 [file 10863_2024_10022_MOESM1_ESM.tif]

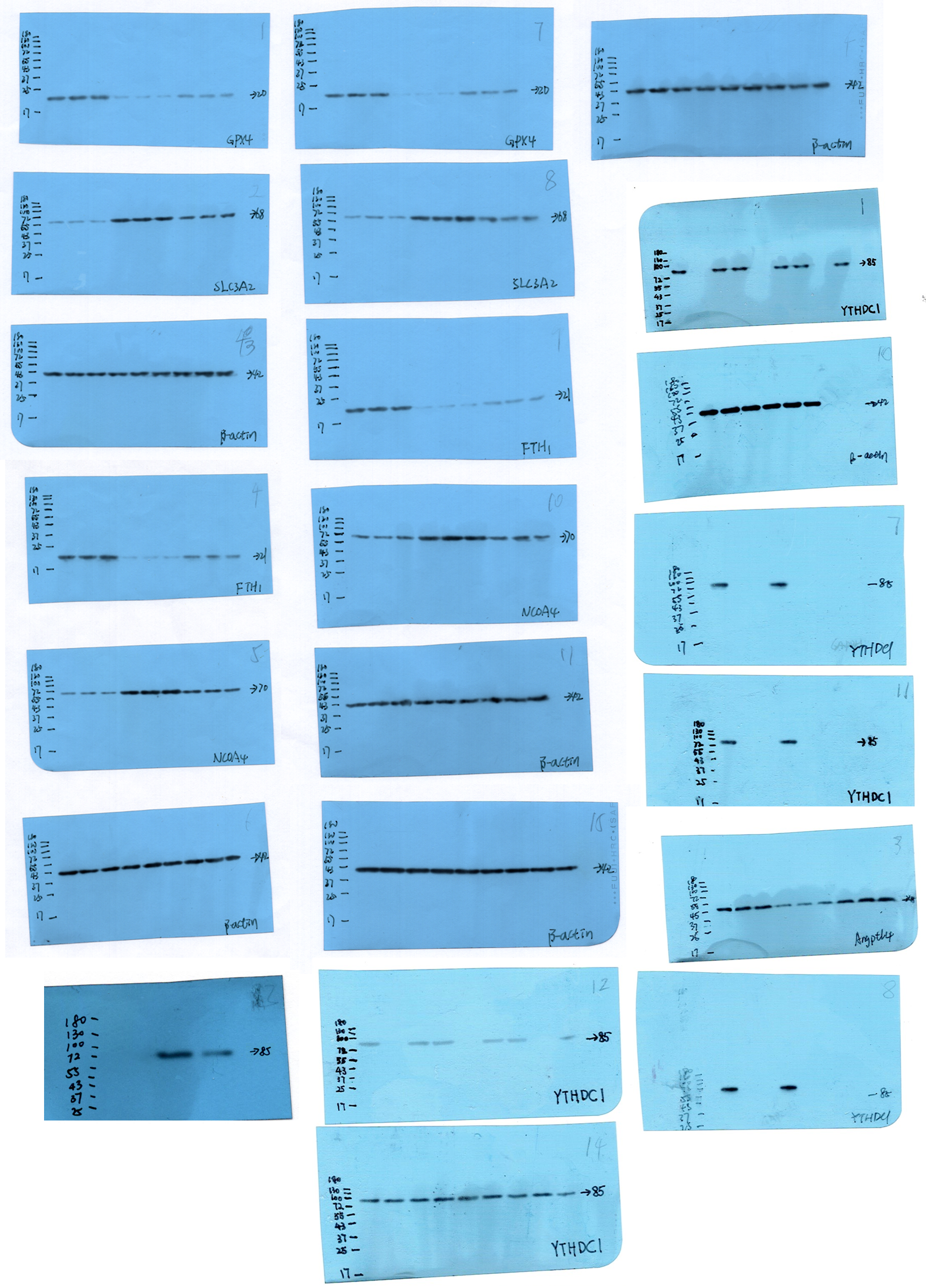

Supplement: Supplementary file 2 — Supplementary Material 2 [file 10863_2024_10022_MOESM2_ESM.tif]

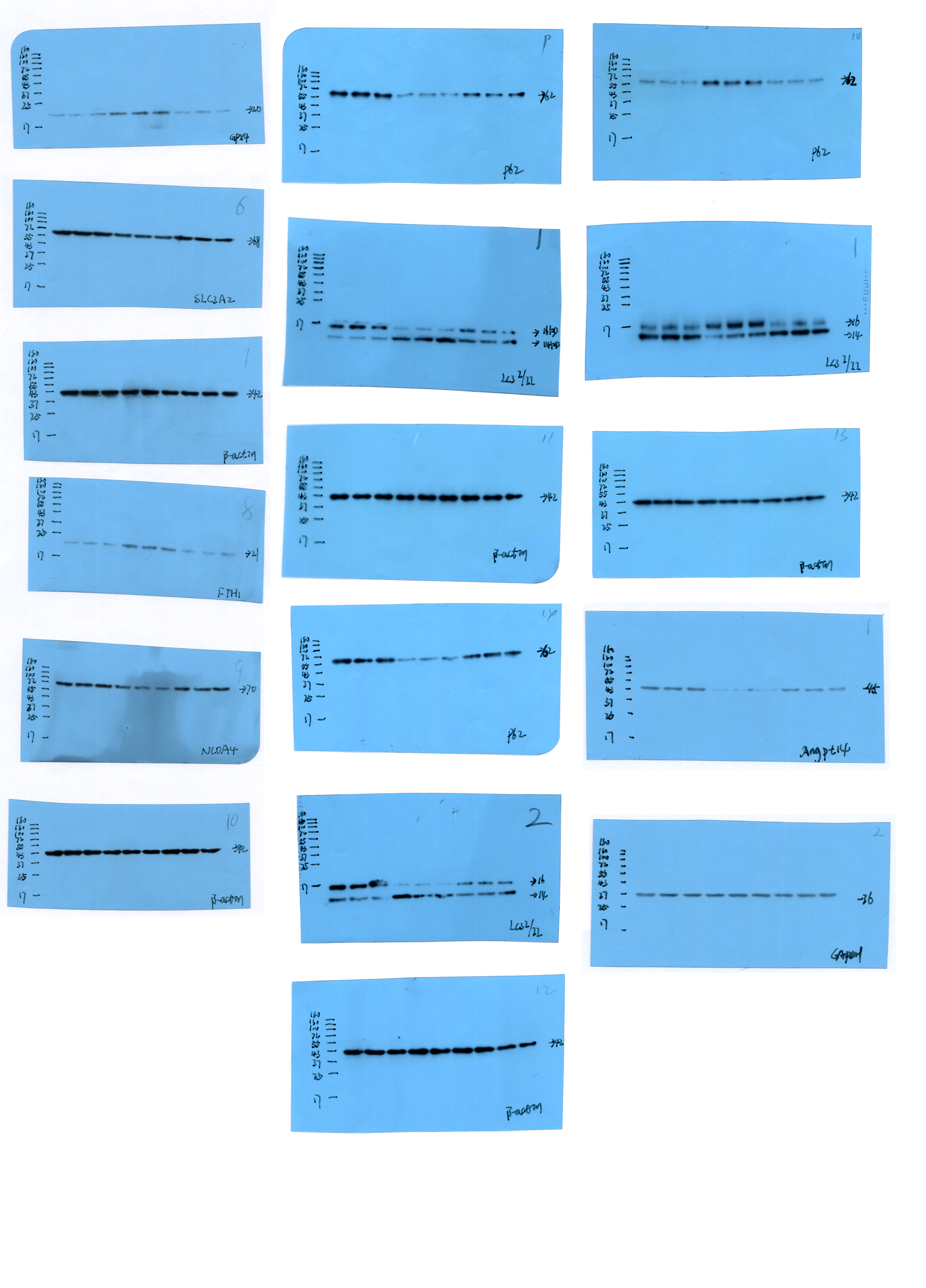

Supplement: Supplementary file 3 — Supplementary Material 3 [file 10863_2024_10022_MOESM3_ESM.tif]
